# Hydrogen Sulfide Regulates Irisin and Glucose Metabolism in Myotubes and Muscle of HFD-Fed Diabetic Mice

**DOI:** 10.3390/antiox11071369

**Published:** 2022-07-14

**Authors:** Rajesh Parsanathan, Sushil K. Jain

**Affiliations:** Department of Pediatrics and Center for Cardiovascular Diseases and Sciences, Louisiana State University, Health Sciences Center-Shreveport, Shreveport, LA 71130, USA

**Keywords:** hydrogen sulfide, l-cysteine, CSE, FNDC5, Irisin, PGC-1α, GLUT4, T2DM, obesity, muscle

## Abstract

Irisin, a novel myokine, is secreted by the muscle following proteolytic cleavage of fibronectin type III domain containing 5 (FNDC5) and is considered a novel regulator of glucose homeostasis. Cystathionine γ-lyase (CSE) produces hydrogen sulfide (H_2_S) and is involved in glucose homeostasis. We examined the hypothesis that H_2_S deficiency leads to decreased FNDC5 and irisin secretion, and thereby alters glucose metabolism. High-fat diet-fed mice exhibited elevated blood glucose and significantly reduced levels of CSE, H_2_S, and PGC-1α, with decreased FNDC5/irisin levels and increased oxidative stress in the muscle compared with those of normal diet-fed mice (control). High glucose or palmitate decreases CSE/PGC-1α/FNDC5 levels and glucose uptake in myotubes. Inhibitors (propargylglycine and aminooxyacetate) of H_2_S producing enzymes or CSE siRNA significantly decreased levels of H_2_S and FNDC5 along with PGC-1α; similar H_2_S-deficient conditions also resulted in decreased GLUT4 and glucose uptake. The levels of H_2_S, PGC-1α, and FNDC5 and glucose uptake were significantly upregulated after treatment with l-cysteine or an H_2_S donor. Myoblast differentiation showed upregulation of PGC-1α and FNDC5, which was consistent with the increased expression of CSE/H_2_S. These findings suggest that the upregulation of H_2_S levels can have beneficial effects on glucose homeostasis via activation of the PGC-1α/FNDC5/irisin signaling pathway.

## 1. Introduction

Skeletal muscle dysfunction has become a relevant factor in health and metabolic disease. Hormones secreted by skeletal muscle, called myokines [1], play essential roles in regulating glucose homeostasis and lipid metabolism [2]. Skeletal muscle health is crucial because, among other things, a lack of physical activity has contributed to an increase in the global prevalence of diabetes, which has risen from 108 million in 1980 to 422 million in 2014, according to statistics from the Centers for Disease Control and Prevention [3].

Irisin, a novel myokine secreted following proteolytic cleavage of its precursor fibronectin type III domain containing 5 (FNDC5) by the skeletal muscle [4], can regulate glucose homeostasis [5,6]. Irisin secretion occurs in response to peroxisome proliferator-activated receptor γ coactivator-1α (PGC-1α) activation through exercise. Type 2 diabetes mellitus (T2DM) patients show decreased irisin levels, along with other inverse associations with diabetes and its associated complications [7,8,9,10,11]. Serum irisin levels are closely related to those of HOMA-β under conditions of normal glucose tolerance, suggesting that irisin may also play an essential role in pancreatic β-cell function [12]. Decreased circulating irisin concentrations and FNDC5 gene expression have been observed in the muscle of obese T2DM subjects [13].

Hydrogen sulfide (H_2_S) is generated from l-cysteine mainly by cystathionine γ-lyase (CSE), and also by two other enzymes, cystathionine β-synthase (CBS) and 3-mercaptopyruvate sulfurtransferase (3-MST) [14]. Non-enzymatic hydrogen sulfide produced from cysteine is also shown in the blood, and this reaction is catalyzed by iron and vitamin B_6_ [15]. Diabetic patients have lower blood concentrations of H_2_S and l-cysteine (LC) [16,17]. LC undergoes enzymatic breakdown to produce hydrogen sulfide (H_2_S), a gasotransmitter that regulates glucose and lipid homeostasis [18]. It has been reported recently that an association has been observed between the decline in H_2_S levels and the high fructose diet-induced T2DM in rats and high-fat diet-fed mice [18,19,20]. Another recent report suggested that either a systemic increase or decrease in H_2_S levels achieved by pharmacological means causes a reduction in insulin resistance [21], implying that various organ-specific metabolic responses play a role in regulating cardiovascular function, inflammation, insulin resistance, obesity, and glucose metabolism [22,23,24,25,26,27,28,29].

In vitro, in vivo, and human studies from our laboratory and others have shown that a decreased level of H_2_S is associated with high glucose/insulin resistance/diabetes [16,17,18,20,30,31,32,33,34,35,36,37,38,39,40,41,42] and that H_2_S reportedly reduces insulin resistance. Together, these studies suggest that H_2_S exerts control over glucose homeostasis at physiologically relevant concentrations. However, the precise biological effect of both endogenous or exogenous H_2_S on myokine irisin and its contribution to glucose homeostasis is not apparent. No previous study has investigated the impact of H_2_S on the regulation of muscle myokine irisin.

This study is the first to report downregulation of both CSE mediated H_2_S synthesis and FNDC5/irisin, and the subsequent alteration of glucose metabolism in the skeletal muscle of mice fed a HFD. Cell culture studies have shown that either downregulation or upregulation of physiological levels of H_2_S exerted inhibitory/beneficial effects by altering PGC-1α, FNDC5/irisin, and glucose homeostasis. This provides evidence for H_2_S as a new endogenous factor responsible for regulating PGC-1α mediated regulation of irisin and glucose metabolism.

## 2. Materials and Methods

All chemicals and reagents used in the study, which were of molecular and analytical grade, were purchased from Sigma Chemical Co. (St. Louis, MO, USA), unless otherwise mentioned. Antibodies against FNDC5 (ab174833), and PGC-1α (ab54481) were purchased from Abcam. The anti-CTH (WH0001491M), anti-GLUT4 (G4048), and anti-GAPDH HRP (G9295) antibodies were purchased from Sigma Aldrich. Goat anti-mouse HRP (170–6516) was purchased from Biorad and the goat anti-rabbit HRP (12–348) from Millipore.

### 2.1. Animals and Dietary Treatment

C57BL/6J male mice (5 weeks old, 20–24 g; Jackson Laboratory, Bar Harbor, ME, USA) were housed in a temperature-controlled room (22 ± 2 °C) with a 12-h: 12-h light: dark cycle. Animals were acclimatized in the Institutional Animal House for one week. Mice fasted overnight, were weighed and then tested for hyperglycemia by measuring their blood glucose concentrations. Blood glucose was assessed following a tail prick using an Accu-Chek glucometer (Boehringer Mannheim Corp., Indianapolis, IN, USA). Animals were randomly distributed into two groups by computer-generated randomization, and each animal had ad libitum access to water and was fed either a high-fat diet (HFD, Harlan TD.88137, providing 42% calories as fat) or a standard chow feed (control, Harlan TD.08485, providing 5.2% calories as fat) for 16 weeks (*n* = 6, in each group). The detailed composition of these diets is given in a recent publication [43]. This is a reasonable model (dietary-induced insulin resistance) for the human metabolic syndrome condition, which created both fasting hyperglycemia and hyperinsulinemia.

All of the procedures that involved animal handling followed the ethical standards of the institution and were approved by the Institutional Animal Ethical Committee. After 16 weeks, the mice were weighed and sacrificed, blood was collected and plasma isolated after centrifuging the blood in a 4 °C centrifuge at 2000× *g* for 15 min; the plasma was stored at −80 °C until use. Skeletal muscles were collected and cut into pieces adequate for the preparation of homogenates, immediately frozen in liquid nitrogen, and stored at −80 °C until use.

### 2.2. Cell Culture, Gene Silencing, and Treatments

Mouse C_2_C_12_ myoblasts (American Type Culture Collection no. CRL-1772, Manassas, VA, USA) were cultured at 37 °C in an atmosphere of 5% CO_2_ and allowed to differentiate into myotubes [18,44]. CSE/H_2_S deficiency was induced by CSE siRNA (100 nM; 24 h) [18,44] or the chemical inhibitors propargylglycine or aminooxyacetate (100 µM for 6 h). After the transfection procedure, cells were treated with serum and phenol red-free medium for 6 h, similar to the NaHS treatment (described below).

An additional set of experimental myotubes were treated with high glucose (25 mM) or palmitate (0.6 mM) (which mimics diabetic conditions) for 24 h. In another set of experiments, cells were supplemented with either l-cysteine (LC; 300 μM)/Na_2_S (Na_2_S; 20 μM) for 6 h. Alamar Blue reduction bioassay was employed in all the experimental conditions to determine the cell viability.

### 2.3. Analysis of mRNA Expression Using Quantitative PCR and Western Blot Analysis

Quantitative PCR was performed using the TaqMan™ Gene Expression Assays (Applied Biosystems, Waltham, MA, USA) with primer/probe sets CSE (Mm00461247_m1), PGC-1α (Mm01208835_m1), FNDC5 (Mm01181543_m1), GLUT4 (Mm00436615_m1), GAPDH (Mm99999915_g1), Applied Biosystems™, Waltham, MA, USA. In accordance with MIQE guidelines, technical replicates (*n* = 3) and biological replicates (*n* = 4) were included in all of our experiments. The relative amount of fold change mRNA was calculated using the 2^−ΔΔCT^ method with a 7900HT real-time PCR system and software (Applied Biosystems, Waltham, MA, USA), and the results were expressed as relative quantification (RQ).

The tissue/cell homogenates were processed for the immunoblotting studies and the protocol was followed as per our previously published method [18,20,44,45]. Densitometry analyses of Western blots were normalized to GAPDH (ratio).

### 2.4. Hydrogen Sulfide Measurements

Plasma and cell culture supernatant hydrogen sulfide (H_2_S) levels were determined as per the previously published methylene blue method [41,46]. Free sulfide was measured in cells using a specific fluorescent probe, sulfide fluor-7 acetoxymethyl ester (SF_7_-AM) (748110, ALDRICH, St. Louis, MO, USA) [18]. Results were expressed as the fold change with the respective controls ratio of F0/Fi.

### 2.5. Glucose Uptake Assays

The glucose uptake assay was performed using 6-NBDG (Invitrogen, Waltham, MA, USA), a fluorescent analog of 2-deoxyglucose, following the method of Jung et al. [47]. Results were expressed as relative fluorescence units (RFU).

### 2.6. GSH, Protein Carbonyl, MDA, Hydrogen Peroxide, and Irisin Assays

Levels of total GSH from tissues were quantified using a fluorimetric method (#CS1020; Glutathione Assay Kit, Fluorimetric; Sigma, St. Louis, MO, USA). Oxidative stress was assessed by measuring the quantification of protein carbonyls and MDA using a Protein Carbonyl Colorimetric Kit and TBARS Assay kit (Cayman Chemical, Ann Arbor, MI, USA). Amplex™ Red Hydrogen Peroxide/Peroxidase Assay Kit (ThermoFisher Scientific, Grand Island, NY, USA) was used to detect hydrogen peroxide (H_2_O_2_) and expressed as nmol/mg protein. Protocols, as provided in the manufacturer’s instructions, were followed in all the assays using the appropriate controls and standards. Plasma and cell culture supernatant levels of irisin were determined using ELISA kits from Phoenix Pharmaceuticals, Inc. Burlingame, CA, USA (Cat# EK-067-29).

### 2.7. Statistical Analysis

The data were subjected to either Student’s *t*-test to compare the control with the HFD group or one-way analysis of variance (ANOVA), followed by Student’s-Newman–Keul’s (SNK) test to assess the significance between control and experimental groups. The data were considered statistically significant at the level of *p* < 0.05 and expressed as mean ± standard error of the mean (SEM). GraphPad Prism version 8.00 for Windows (GraphPad Software, La Jolla, CA, USA) was used for statistical analysis.

## 3. Results

### 3.1. H_2_S Deficiency Impairs FNDC5/Irisin in the Skeletal Muscle of HFD-Fed Mice

**Figure 1:**
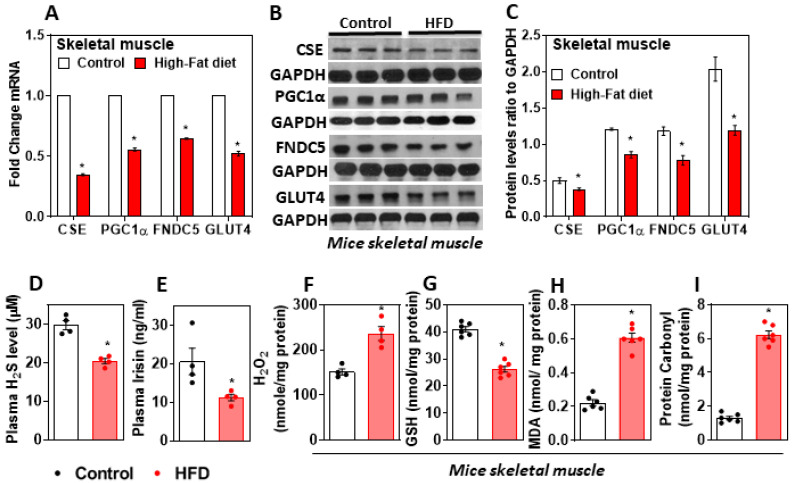
Effect of HFD on muscle CSE-H_2_S, irisin, and oxidative stress. MaleC57BL/6J mice (5 weeks old) were fed either a standard chow diet (control) or a high-fat diet (HFD) for 16 weeks. (**A**) RT-qPCR was performed to assess the level of target genes as indicated (*n* = 6); (**B**) representative Western blot analysis (CSE, PGC-1α, FNDC5, and GLUT4) was performed on total protein extracts (*n* = 3 independent experiments) in the muscle; (**C**) semi-quantitative analysis of the abundance ratio of protein to GAPDH; (**D**) plasma H_2_S (*n* = 4); (**E**) irisin level (*n* = 4); (**F**) hydrogen peroxide [H_2_O_2_] (*n* = 4); (**G**) glutathione level (*n* = 6); (**H**) malondialdehyde(MDA)-lipid peroxidation (*n* = 6); (**I**) protein carbonyl levels in muscle (*n* = 6).The student’s *t*-test was used to compare the control with the HFD group. * *p* ≤ 0.05 was considered significant for a statistical test. Data are expressed as mean ± SEM.

Mice fed a HFD for 16 weeks showed a metabolic phenotype similar to that of obese T2DM human subjects. In the muscle of HFD-fed mice, the expression of CSE decreased along with that of the gene, which encodes for the protein FNDC5, and its cleaved product irisin was downregulated (Figure 1A–C). The genes involved in glucose homeostasis, PGC-1α, and GLUT4, were significantly downregulated in the muscle of HFD-fed mice compared to those of the control group (Figure 1A–C). Furthermore, the levels of plasma H_2_S and irisin decreased significantly, and those of oxidative stress markers, such as H_2_O_2_, MDA, and protein carbonyl, increased in HFD-fed mice muscle compared to those of controls (Figure 1D–I). This suggests that a decreased status for the antioxidants GSH, H_2_S, and excess oxidative stress in the muscle of diabetic mice blunts the expression of FNDC5 and the levels of myokine irisin.

### 3.2. Treatment with High Glucose and Palmitate Affects H_2_S Levels and FNDC5/Irisin Expression and Secretion in In Vitro myotubes

**Figure 2:**
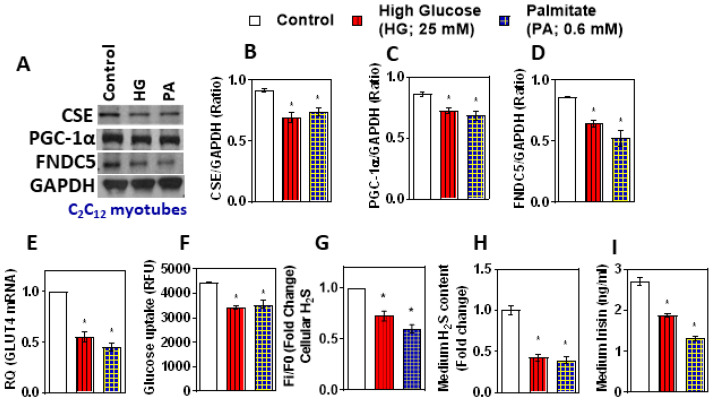
Effect of treatment with high glucose and palmitate on CSE-H_2_S, PGC-1α, FNDC5 (irisin), and glucose uptake in C_2_C_12_ mouse myotubes. Differentiated myotubes treated with high glucose (25mM) or palmitate (0.6mM) for 24 h. Mannitol was used as an osmolarity control. (**A**) Western blot analysis (CSE, PGC-1α, and FNDC5) was performed on total protein extracts (*n* = 3 independent experiments) in mouse myotubes; (**B**–**D**) semi-quantitative analysis of the abundance ratio of protein to GAPDH. (**E**) RT-qPCR was performed to assess the level of the GLUT4 gene, as indicated (*n* = 3). (**F**) Glucose uptake; (**G**) levels of intracellular H_2_S; (**H**) cell culture medium H_2_S; (**I**) cell culture medium irisin levels. One-way ANOVA followed by SNK (Student’s Newman–Keul’s) means comparison was performed between the control and treatment groups. * *p* ≤ 0.05 was considered significant for a statistical test. Data are expressed as mean ± SEM.

Differentiated myotubes were used to investigate expression and secretion of FNDC5/irisin in an in vitro condition, which is devoid of the neuro humoral milieu of an integral body. Treatment with high glucose (25mM) or palmitate (0.6mM) (which mimics diabetic conditions) for 24 h decreased CSE, PGC-1α, and FNDC5 protein levels in differentiated mouse myotubes (Figure 2A–D); the levels of irisin in the condition medium also significantly decreased (Figure 2I). Furthermore, in vitro, the diabetic conditions significantly decreased the levels of GLUT4 mRNA and glucose uptake, along with cellular and medium H_2_S levels (Figure 2E–H). From these data, it is inferred that diabetic conditions may be directly triggered to downregulate the CSE-H_2_S system and irisin.

### 3.3. Inhibition of H_2_S Production Reduces Irisin and Glucose Uptake

**Figure 3:**
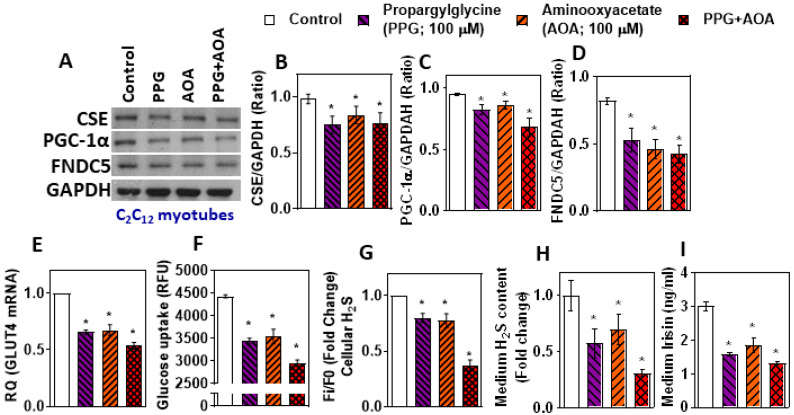
Effect of H_2_S producing enzyme inhibitors (propargylglycine or aminooxyacetate) on CSE-H_2_S, PGC-1α, FNDC5 (irisin), and glucose uptake in C_2_C_12_ mouse myotubes. Differentiated myotubestreated with propargylglycine (PPG; 100 µM) oraminooxyacetate (AOA; 100 µM) or a combination of both for 6 h. (**A**) Western blot analysis (CSE, PGC-1α, and FNDC5) was performed on total protein extracts (*n* = 3 independent experiments) in mouse myotubes; (**B**–**D**) semi-quantitative analysis of the abundance ratio of protein to GAPDH. (**E**) RT-qPCR was performed to assess the level of the GLUT4 gene, as indicated (*n* = 3). (**F**) Glucose uptake; (**G**) levels of intracellular H_2_S; (**H**) cell culture medium H_2_S; (**I**) cell culture medium irisin levels. One-way ANOVA followed by SNK (Student’s Newman–Keul’s) means comparison was performed between the control and treatment groups. * *p* ≤ 0.05 was considered significant for a statistical test. Data are expressed as mean ± SEM.

Myotubes treated with pharmacological inhibitors of H_2_S producing enzymes (propargylglycine or aminooxyacetate; 100 µM for 6 h) showed decreased levels of H_2_S and FNDC5 along with PGC-1α (Figure 3A–D); similar H_2_S-deficient conditions also caused decreases in both GLUT4 and glucose uptake (Figure 3E,F). The levels of irisin in the condition medium decreased significantly along with inhibition of H_2_S (Figure 3G–I). These data imply that inhibition of the H_2_S system can affect irisin and glucose uptake in myotubes.

### 3.4. CSE Knockdown Affects the PGC-1α Mediated Uptake of Irisin, GLUT4, and Glucose

**Figure 4:**
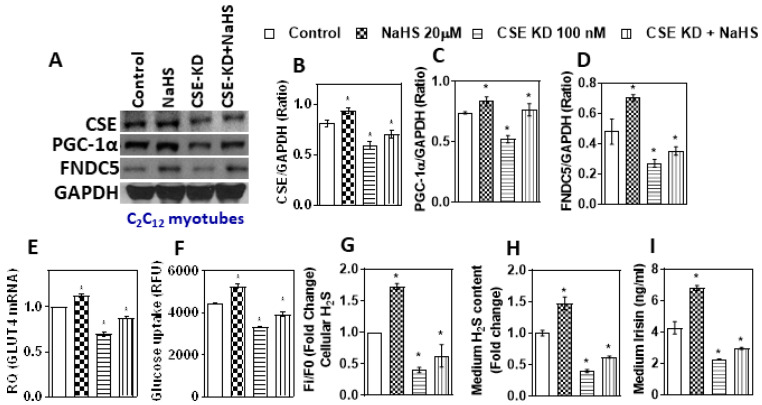
Effect of sodium hydrosulfide (a donor of H_2_S) treatment on CSE/H_2_S-deficient myotubes and the levels of CSE-H_2_S, PGC-1α, FNDC5 (irisin), and glucose uptake. CSE/H_2_S-normal (scrambled siRNA) and CSE/H_2_S-deficient myotubes (100 nM CSE siRNA) treated with sodium hydrosulfide (NaHS, 20 µM). A scrambled siRNA nonspecific RNA duplex with no sequence homology with any of the genes served as a control. (**A**) Western blot analysis (CSE, PGC-1α, and FNDC5) was performed on total protein extracts (*n* = 3 independent experiments) in mouse myotubes; (**B**–**D**) semi-quantitative analysis of the abundance ratio of protein to GAPDH. (**E**) RT-qPCR was performed to assess the level of the GLUT4 gene, as indicated (*n* = 3). (**F**) Glucose uptake; (**G**) levels of intracellular H_2_S; (**H**) cell culture medium H_2_S; (**I**) cell culture medium irisin levels. One-way ANOVA followed by SNK (Student’s Newman–Keul’s) means comparison was performed between the control and treatment groups. * *p* ≤ 0.05 was considered significant for a statistical test. Data are expressed as mean ± SEM.

The direct effect of CSE-H_2_S deficiency status on PGC-1α, FNDC5/irisin, and GLUT4 and glucose uptake was assessed using in vitro CSE siRNA (100 nM; 24 h) experiments on mouse myotubes. CSE knockdown (KD) cells showed decreased CSE expression and H_2_S content (Figure 4A,B,G). PGC-1α and FNDC5 expression were also attenuated in CSE KD cells compared to that in control cells (Figure 4A,C,D). Additionally, similar decreasing trends were observed for irisin, GLUT4 mRNA, and glucose uptake in the condition medium of CSE KD cells (Figure 4E,F,H,I). These results suggest that the status of H_2_S may have a direct effect on the muscle myokine irisin.

### 3.5. H_2_S Donors/Precursor (NaHS/Na_2_S/LC) Positively Regulate PGC-1α, Irisin, and GLUT4 Mediated Glucose Uptake in Myoblasts

**Figure 5:**
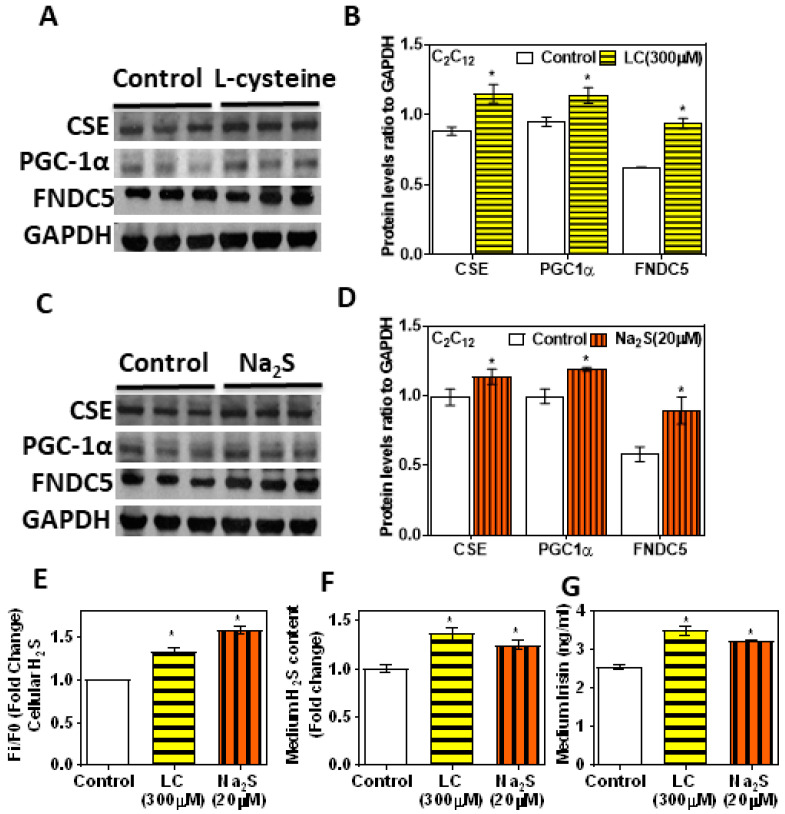
Effect of treatment with l-cysteine and sodium sulfide (a donor of H_2_S) on C_2_C_12_ mouse myotube CSE-H_2_S, PGC-1α, and FNDC5 (irisin). Differentiated myotubes treated with either l-cysteine (LC; 300 µM) or sodium sulfide (Na_2_S; 20 µM) for 6 h. (**A**,**C**) Western blot analysis (CSE, PGC-1α, and FNDC5) was performed on total protein extracts (*n* = 3 independent experiments) in mouse myotube; (**B**,**D**) semi-quantitative analysis of the abundance ratio of protein to GAPDH. (**E**) Levels of intracellular H_2_S; (**F**) cell culture medium H_2_S; (**G**) cell culture medium irisin level. The Student’s *t*-test was used to compare the controls with the LC/Na_2_S group. One-way ANOVA followed by SNK (Student’s Newman–Keul’s) means comparison was performed between the control and treatment groups. * *p* ≤ 0.05 was considered significant for a statistical test. Data are expressed as mean ± SEM.

The CSE KD cells supplemented with an H_2_S donor showed a beneficial effect over the H_2_S system and FNDC5/irisin levels (Figure 4). The levels of CSE, H_2_S, PGC-1α, and FNDC5 and glucose uptake were significantly upregulated after treatment with LC (300 µM) or H_2_S donor (NaHS/Na_2_S; 20 µM) for 6 h (Figure 5A–F). The levels of irisin in the condition medium also significantly increased in the LC and H_2_S donor groups compared to those in the control group (Figure 5G). Cell viability was not affected under any of these conditions. This suggests that H_2_S donors/precursors may have beneficial effects over myokine irisin and glucose homeostasis.

### 3.6. Myoblast Differentiation Synergistically Upregulates CSE-H_2_S and the PGC-1α-FNDC5/Irisin Pathway

**Figure 6:**
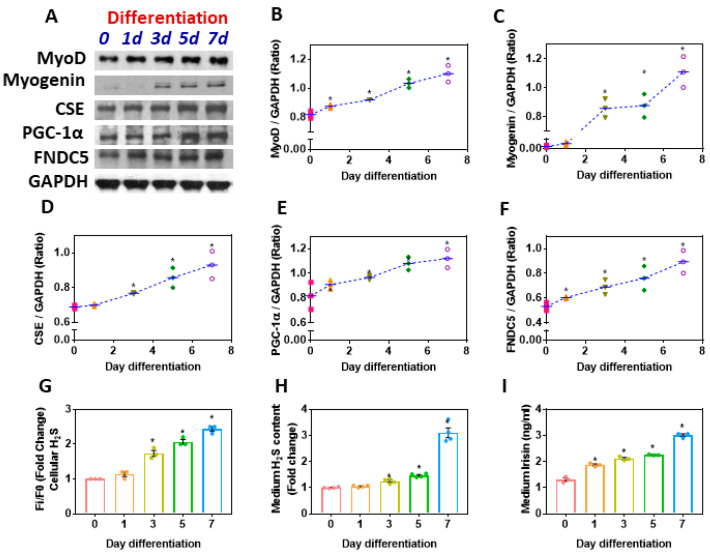
Effect of the myogenic commitment to differentiate C_2_C_12_ myoblasts into myotubes on the levels of CSE-H_2_S, PGC-1α, and FNDC5 (irisin). C_2_C_12_ cells grown to confluence and differentiated into myotubes were cultured in DMEM with 2% horse serum for up to 7 days. (**A**) Western blot analysis to monitor the expression of myogenic factors (MyoD, myogenin) and CSE, PGC-1α, and FNDC5 was performed on total protein extracts (*n* = 3 independent experiments) during C_2_C_12_ cell differentiation at days 0, 1, 3, 5, and 7 of myogenesis; (**B**–**F**) semi-quantitative analysis of the abundance ratio of protein to GAPDH. (**G**) Intracellular H_2_S levels; (**H**) cell culture medium H_2_S; (**I**) cell culture medium irisin levels. One-way ANOVA followed by SNK (Student’s Newman–Keul’s) means comparison was performed between the control and treatment groups. * *p* ≤ 0.05 was considered significant for a statistical test. Data are expressed as mean ± SEM.

Myogenesis is affected during metabolic disorders, such as obesity and diabetes mellitus. Myoblast differentiation shows significant upregulation of PGC-1α and FNDC5, which was consistent with the increased expression of CSE-H_2_S, along with levels of myogenic markers (MyoD and myogenin) (Figure 6A–I). These findings suggest that the upregulation of physiological levels of H_2_S can have beneficial effects on muscle glucose homeostasis via the PGC-1α/FNDC5/irisin pathway.

## 4. Discussion

H_2_S has been shown to elicit a variety of biological effects and has been gaining acceptance as a signaling molecule that may mediate protection from various metabolic disorders [23,24,25,26,27,28,29,48]. Experimental and clinical studies reveal the importance of irisin in improving insulin sensitivity, pancreatic β cell function, and the browning of white adipose tissue and associated metabolic disorders [49]. This study provides novel evidence that circulating levels of irisin/tissue FNDC5 can be regulated by hydrogen sulfide. The decrease in H_2_S (cystathionine γ-lyase-mediated) is positively associated with muscle FNDC5 expression and the plasma irisin level in HFD-fed obese diabetic mice. This is consistent with recent studies in metabolic syndrome/obese/diabetic subjects showing decreased blood levels of H_2_S [18,19,20], and irisin [7,8,9].

This study demonstrates that a HFD causes a lower level of circulating H_2_S and irisin, with decreased expression of the irisin precursor FNDC5 in the muscle of HFD-fed mice. H_2_S positively regulates glutathione biosynthesis and inhibits oxidative stress in myotubes [18,44]. It is well recognized that diabetes induces oxidative stress that causes subchronic immuno-inflammatory conditions. ATB-346, a hydrogen sulphide (H_2_S)-releasing anti-inflammatory and analgesic drug, significantly boosted H_2_S levels and inhibited cyclooxygenase activity in a phase 2B study, with minimal gastrointestinal effects [50]. Multiple sclerosis is associated with decreased expression of the H2S-producing enzyme 3-mercaptopyruvate-sulfurtransferase, whereas H_2_S donor GYY4137 upregulates tolerogenic pathways [51]. H_2_S and its donors have been shown to be effective in preclinical animal models of autoimmune, acute, and chronic inflammatory disorders [52]. In this study, decreased cystathionine γ-lyase expression leads to H_2_S deficiency, which induces oxidative stress in myotubes exposed to high glucose or palmitate (which mimics diabetic conditions) and in the muscle of HFD-fed mice. Furthermore, deficiency of H_2_S impairs expression of PGC-1α, GLUT4, and glucose uptake in the muscle. H_2_S deficiency induced in myotubes by the pharmacological inhibitors (PPG or AOA) or CSE gene silencing reduced levels of PGC-1α, irisin, GLUT4, and glucose uptake. This is in line with data from the muscle of HFD-fed mice. It should also be noted that our data demonstrate that myoblast differentiation synergistically upregulates CSE-H_2_S, PGC-1α, and FNDC5/irisin, along with myogenic markers (MyoD and myogenin). These results suggest the novel physiological role played by the H_2_S system during myogenesis, which can directly affect the myokine irisin and impair glucose disposal in myotubes. This study makes the novel observation that supplementation with H_2_S donors or precursors beneficially regulates CSE-H_2_S, PGC-1α, and FNDC5/irisin. This suggests that circulating levels of H_2_S have a potentially significant effect on irisin and glucose metabolism.

H_2_S as a signaling molecule may trigger various biochemical pathways and activate PGC-1α, which upregulates irisin and favors glucose uptake. H_2_S promotes stimulation of adenosine triphosphate-sensitive potassium (K_ATP_) channels [53,54]. NaHS (an H_2_S donor) increases intracellular calcium [Ca^2+^]i through NMDA receptor, L-type, and T-type calcium channels, which may mobilize a movement of calcium from the ER into the cytosol via the ryanodine receptor [55,56]. Exercise induces calcium influx and activates transcriptional co-factor PGC-1α, which regulates FNDC5 expression/release of irisin from myocytes [4,57]. It has been suggested that an exercise-independent form of H_2_S-mediated Ca^2+^ influx may activate PGC-1α and induce the expression of FNDC5. It is possible that exogenous H_2_S increases PGC-1α. It has been shown that NaHS also increased mitochondrial biogenesis by upregulation of the expression of PGC-1α in the rat liver [58]. Sirtuin 1 activates PGC-1α via deacetylation [59], and AMPK activates PGC-1α via phosphorylation [60]; recently, it was shown in primary hepatocytes that PGC-1α activity was enhanced via S-sulfhydration with no change in acetylation modification [61]. H_2_S signals via protein S-sulfhydration as a physiological post-transcriptional modification of cysteine residues in the target protein that leads to enhanced protein function [62]. Indeed, future mechanistic studies of how H_2_S regulates PGC-1α in the maintenance of FNDC5/irisin and glucose homeostasis, whether by an exercise-independent increase in Ca^2+^ influx or by protein S-sulfhydration and activation of PGC-1α, would be interesting.

## 5. Conclusions

**Figure 7:**
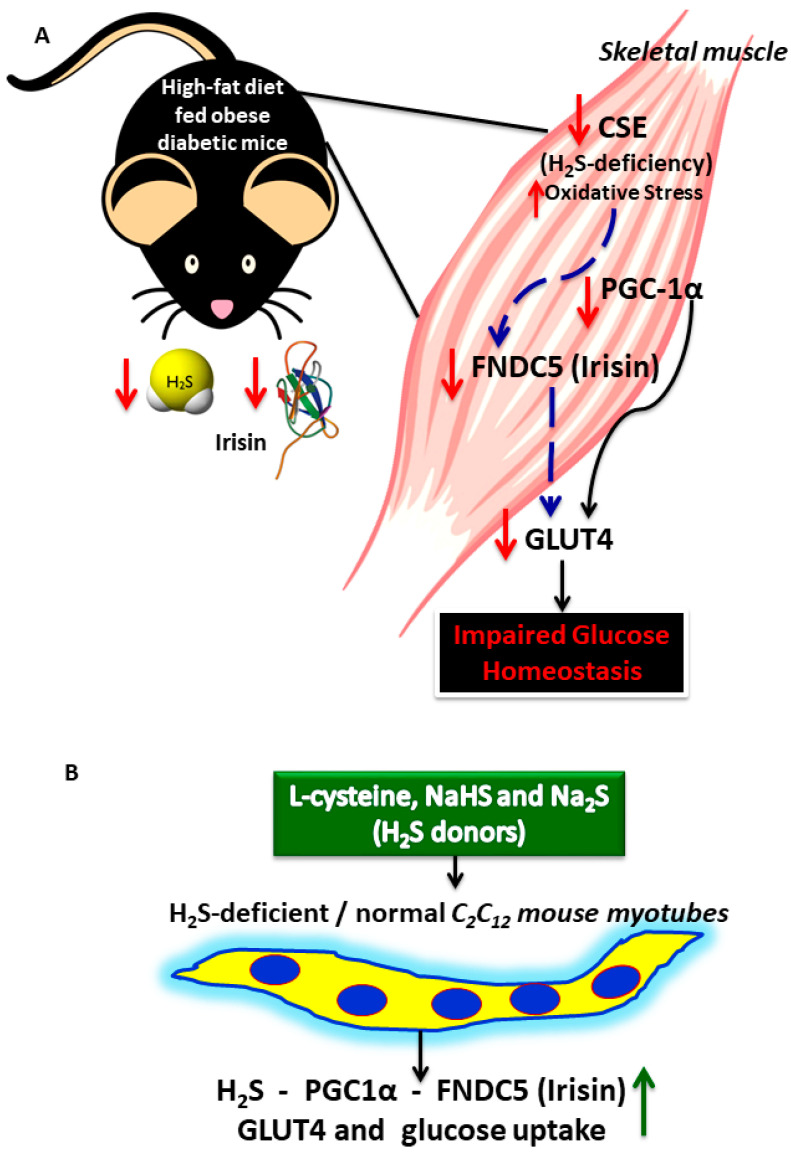
Schematic presentation of the proposed mechanism by which an HFD-induced deficiency of the CSE/H_2_S system alters PGC-1α, FNDC5 (irisin, a muscle myokine), and glucose homeostasis in the muscle (**A**), and how supplementation with l-cysteine, NaHS, or Na_2_S (H_2_S donors) increases irisin and GLUT4 mediated glucose uptake in C_2_C_12_ mouse myotubes (**B**).

H_2_S deficiency alters the PGC-1α/FNDC/irisin signaling pathway and glucose homeostasis in the muscle of HFD-fed obese diabetic mice (Figure 7A). Supplementation with l-cysteine (an H_2_S precursor), NaHS, or Na_2_S (H_2_S donors) increases levels of irisin and positively regulates GLUT4 mediated glucose uptake in C_2_C_12_ mouse myotubes (Figure 7B). These findings demonstrate that the upregulation of physiological levels of H_2_S can have beneficial effects on irisin secretion and glucose homeostasis via the PGC-1α signaling pathway. The understanding and validation of the mechanisms by which H_2_S supplementation improves glycemia should support the design of clinical intervention using novel molecules (containing sulfide and cysteine moieties) to improve glucose metabolism. This study suggests a novel potential role for H_2_S donors as adjuvant therapy in the treatment of metabolic complications in diabetes.

## Data Availability

Data is contained within this article.
